# The complete chloroplast genome of *Tainia dunnii* (Orchidaceae): genome structure and evolution

**DOI:** 10.1080/23802359.2019.1693935

**Published:** 2019-12-10

**Authors:** Tai-Xiang Xie, Xia Yu, Qing-Dong Zheng, Shan-Hu Ma, Zhong-Jian Liu, Ye Ai

**Affiliations:** Key Laboratory of National Forestry and Grassland Administration for Orchid Conservation and Utilization at College of Landscape Architecture, Fujian Agriculture and Forestry University, Fuzhou, China

**Keywords:** Chloroplast genome, phylogenetic analysis, Orchidaceae, *Tainia dunnii*

## Abstract

*Tainia dunnii* is a terrestrial orchid with high ornamental value. Herein, we assembled the complete chloroplast genome of *Tainia dunnii* by next-generation sequencing technologies. The complete chloroplast genome sequence of *Tainia dunnii* is 158,305 base pairs (bp) in length, including a pair of inverted repeat regions (IRs, 25,244 bp), one large single-copy region (LSC, 86,819 bp), one small single-copy region (SSC, 20,998 bp). Besides, the complete chloroplast genome contains 136 genes in total, including 88 protein-coding genes, 38 tRNA genes, and 8 rRNA genes. Phylogenetic analysis showed that *Tainia dunnii* has the closest relationship with *Calanthe davidii* and *Calanthe triplicata*. Our study lay a foundation for further research of *Tainia dunnii*.

*Tainia*, a genus, belongs to the Orchidaceae family, contains approximately 32 species, distributed north to China and Japan, and south to New Guinea and the Pacific Islands (Wu and Raven 2009). *Tainia dunnii* is a terrestrial orchid widely distributed in southern part of China, such as Jiangxi, Fujian, and Guangdong Province (Wu and Raven 2009). *Tainia dunnii* is often planted as potted plants and has high ornamental value with beautiful flowers and long inflorescences. However, there are very few studies on the *Tainia dunnii*, which greatly limit the development and utilization of *Tainia dunnii*. In this study, we assembled the complete chloroplast genome of *Tainia dunnii*, hoping to lay a foundation for further research.

The samples of *Tainia dunnii* were collected from Qinglong waterfall scenic area (25°46′22.18″N, 118°57′50.14″E), in Yongtai city, Fujian province, China, and the specimens are kept in the Herbarium of Fujian Agriculture and Forestry University (specimen code FAFU01867).

**Figure 1.**
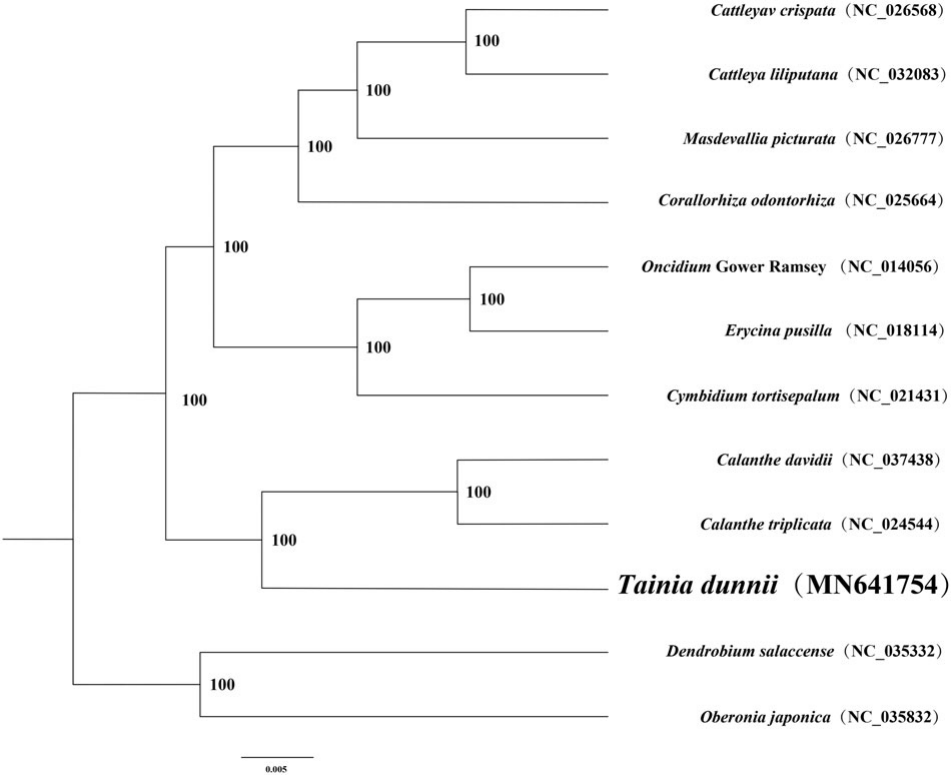
A phylogenetic tree was constructed based on 12 complete chloroplast genome sequences of Orchidaceae. All the sequences were downloaded from NCBI GenBank.

High-quality genomic DNA of *Tainia dunnii* was extracted from leaves by TIANGEN plant genomic DNA kit, and sequenced by the BGISEQ-500 platform. With the chloroplast genome of *Calanthe davidii* (GenBank accession number NC.037438) as the reference sequences, we assembled the complete chloroplast genome from the clean reads by the GetOrganelle pipe-line (Jin et al. 2018), and then annotated the new sequences using the Geneious R11.15 (Kearse et al. 2012). Finally, a complete chloroplast genome of *Tainia dunnii* was obtained and submitted to Genbank (accession number MN641754).

The complete chloroplast genome of *Tainia dunnii* was 158,305 bp in length, consisting of two inverted repeat (IR) regions of 25,244 bp, a large single-copy (LSC) region of 86,819 bp, a small single-copy (SSC) region of 20,998 bp. Besides, the complete chloroplast genome has 136 genes in total, including 88 protein-coding genes, 38 tRNA genes, and 8 rRNA genes. In addition, the overall GC content of the genome was 37.2%.

In order to confirm the phylogenetic position of *Tainia dunnii*, a maximum likelihood analysis was performed by MEGA 6.0 (Tamura et al. 2013) with 1000 bootstrap replicates (Minh et al. 2013; Chernomor et al. 2016) based on 12 complete chloroplast genomes of Orchidaceae species, including *Cattleya crispata* (NC.026568), *Cattleya liliputana* (NC.032083), *Masdevallia picturata* (NC.026777), *Corallorhiza odontorhiza* (NC.025664), *Oncidium* Gower Ramsey (NC.014056), *Erycina pusilla* (NC.018114), *Cymbidium tortisepalum* (NC.021431), *Calanthe davidii* (NC.037438), *Calanthe triplicata* (NC.024544), *Dendrobium salaccense* (NC.035332), and *Oberonia japonica* (NC.035832). The results showed that *Tainia dunnii* was related to *Calanthe davidii* and *Calanthe triplicata* (Figure 1).
